# Risk Knowledge in Relapsing Multiple Sclerosis (RIKNO 1.0) - Development of an Outcome Instrument for Educational Interventions

**DOI:** 10.1371/journal.pone.0138364

**Published:** 2015-10-02

**Authors:** C. Heesen, J. Kasper, K. Fischer, S. Köpke, A. Rahn, I. Backhus, J. Poettgen, L. Vahter, J. Drulovic, A. Van Nunen, Y. Beckmann, K. Liethmann, A. Giordano, G. Fulcher, A. Solari, AutoMS-group

**Affiliations:** 1 Institute of Neuroimmunology and MS Research (INIMS), University Medical Center Eppendorf, Hamburg, Germany; 2 Dep. of Neurology, University Medical Center Eppendorf, Hamburg, Germany; 3 Department of Health and Caring Sciences, Faculty of Health Sciences, University of Tromsø, Tromsø, Norway; 4 Institute for Social Medicine and Epidemiology, University of Lübeck, Lübeck, Germany; 5 Unit of Health Sciences and Education, MIN Faculty, University of Hamburg, Hamburg, Germany; 6 Department of Neurology, West-Tallinn Central Hospital, Tallinn, Estonia; 7 Institute of Neurology, Clinical Center of Serbia, University of Belgrade, Belgrade, Serbia; 8 National MS-Centrum, Melsbroek, Belgium; 9 Department of Neurology, Ataturk Training and Research Hospital, Faculty of Medicine, Izmir, Turkey; 10 Unit of Neuroepidemiology, Foundation IRCCS Neurological Institute C. Besta, Milan, Italy; 11 Psychlinx, Sydney, Australia; University of Muenster, GERMANY

## Abstract

**Background:**

Adequate risk knowledge of patients is a prerequisite for shared decision making but few attempts have been made to develop assessment tools. Multiple Sclerosis (MS) is a chronic inflammatory disease of young adults with an increasing number of partially effective immunotherapies and therefore a paradigmatic disease to study patient involvement.

**Objective/methods:**

Based on an item bank of MS risk knowledge items and patient feedback including perceived relevance we developed a risk knowledge questionnaire for relapsing remitting (RR) MS (RIKNO 1.0) which was a primary outcome measure in a patient education trial (192 early RRMS patients).

**Results:**

Fourteen of the RIKNO 1.0 multiple-choice items were selected based on patient perceived relevance and item difficulty indices, and five on expert opinion. Mean item difficulty was 0.58, ranging from 0.14 to 0.79. Mean RIKNO 1.0 score increased after the educational intervention from 10.6 to 12.4 (p = 0.0003). Selected items were particularly difficult (e.g. those on absolute risk reductions of having a second relapse) and were answered correctly in only 30% of the patients, even after the intervention.

**Conclusion:**

Despite its high difficulty, RIKNO 1.0 is a responsive instrument to assess risk knowledge in RRMS patients participating in educational interventions.

## Introduction

Patient information and knowledge is a prerequisite of informed decision making [1,2]. Therefore, information and knowledge are essential for patient participation in health care. Risk knowledge in the context of a medical condition encompasses concepts such as disease determinants, inheritance, prognosis, benefits and harms of treatments [3]. The communication of risk using probabilities implies information on unknown issues. Consideration has to be given explicitly to stochastic uncertainties, uncertainty of clinical judgements and uncertainty due to lacking evidence [4].

Multiple sclerosis (MS) is a chronic inflammatory disease of young adults with many scientific uncertainties from making a diagnosis, having a prognostic estimate to effects of immunotherapies [5]. Risk knowledge is increasingly relevant in MS as the number of treatment options increases [5]. The risk of adverse events is especially important for more efficacious treatments, the case of progressive multifocal leucencephalopathy (PML) in natalizumab treatment being paradigmatic in this respect. Moreover, risk might be highly dynamic and risk assessment needs regular updating. PML risk in natalizumab treated patients was 1 in 1000 when the drug was marketed again in 2007, but nowadays it is 3.96 in 1000 (www.tysabri.de, state 3.6.2015). The recently licensed alemtuzumab also has substantial risk with approximately 30 out of 100 of patients developing treatment-induced autoimmune diseases [6]. With the newer second line treatments, regulatory authorities and pharmaceutical companies have started to implement MS risk management plans and treatment registries. Risk communication and management need to address not only adverse events of ongoing therapies as in the case of natalizumab, but also possible future risks e.g. of developing leukaemia years after mitoxantrone exposure, or autoimmune diseases after alemtuzumab exposure [7, 8].

Risk knowledge is important both in the clinical setting, where it is necessary to patients to make informed decisions, as well as in research, as an outcome measure to assess the efficacy of patient education programs. However, no rigorously developed and validated risk knowledge instrument for people with MS (PwMS) exists. Giordano et al. [9] developed a 25-item MS knowledge questionnaire (MSKQ) to be applied in the MS peri-diagnostic period by PwMS and MS health professionals, but not especially focussing on risks and uncertainties. The MSKQ was a co-primary outcome measure in a randomized controlled trial (RCT, [10]) and in a late phase controlled study [11] on an information aid for newly-diagnosed PwMS.

In 2004 [12] we published a 19-item risk knowledge questionnaire (RIKNO) focussing on MS immunotherapies, based on the concept of evidence-based patient information (EBPI, [13]). The tool was completed by 200 PwMS, half of whom had primary progressive (PP) course: on average, seven out of the 19 questions were answered correctly, with relapsing remitting (RR) PwMS diagnosed within the previous year providing a higher number of correct answers (8.2 on average) compared to RR PwMS diagnosed > 1 year (7.2 correct answers) and PPMS (5.2 correct answers; p = 0.001). An educational intervention in RRMS improved risk knowledge (as assessed with RIKNO) and patient ability to deal with risk calculation tasks [14]. Poor risk knowledge in this study population might have been caused by the recruitment strategy, which did not address only PwMS at the point of making a treatment decision. Relevance of the risk knowledge item pool in this first study [12] might also be limited because of a lack of patient input in the process of defining essential knowledge items.

We thus developed a new tool, named risk knowledge 1.0 (RIKNO 1.0), specific for PwMS with early RRMS, who should be in the most suitable condition for an MS education program. In the development of the instrument, we gave priority to ensuring a strong involvement of patients and a balance of patient and expert judgement of relevance. RIKNO 1.0 was based on the current understanding of framing risk communication in medicine [15]. It was an outcome measure in the Patient Education ProgrAmme on DIagnosis, Prognosis and early treatment of MS (PEPADIP) study, a RCT on patient education in early RR PwMS [16]. This paper summarizes the developmental process of RIKNO 1.0.

## Methods

### Development of an item bank

In a first step, a pool of 197 items referring to five categories of MS risk knowledge was generated by a panel of experts (two MS neurologists, one health scientist, one psychologist, one nurse) based on literature review, clinical expertise and on the previously developed questionnaire [12]. The five categories defined by the experts included general MS issues, diagnosis, prognosis, treatment, and evidence-based medicine.

### Item selection and questionnaire construction

The 197 items pool (items 1–192 having yes/no answers, and items 193–197 multiple-choice answers) was sent to 120 PwMS randomly selected from the database (5000 PwMS) of the MS Day Hospital at the UMC Hamburg Eppendorf. Participants' knowledge was assessed from the number of correct answers. Moreover, they were asked to rate each item relevance on a 4-level Likert scale, ranging from "not relevant” (0) to "highly relevant" (3). Item difficulty corresponded to the proportion of participants providing the correct answer. Here item difficulty of “1” would mean that everyone did give the correct answer, while item difficulty of “0” would mean no one. Based on both information, item difficulty and patient relevance, the expert panel performed a systematic selection of items from the original pool. Four groups of PwMS with potentially varying needs were identified, as follows: early RRMS (< 2 years from diagnosis), RRMS, secondary progressive MS (SPMS) and primary progressive MS (PPMS). Corresponding core sets of relevant risk-knowledge with good measurement properties (for definition see below in statistical analysis section) were devised. Based on the concept that early RR MS patients based on a more recent diagnosis and the available therapeutic options may have highest knowledge and interest in risks which has been shown in earlier work [12], further validation studies focussed on this patient group. Observational data and notes from the qualitative interviews in a pilot study as well as quantitative findings from this study (see below for details) were considered by the expert panel. Further refinement of each of the 19 items was decided by the expert panel by consensus. The resulting questionnaire (RIKNO 1.0) has a total score obtained by summing the number of correct answers and ranges from 0 to 19. Minimum scores are defined by the lowest number of correct replies in a given cohort while maximum scores refer to the highest number of correct items.

### Pilot study

Participants of four pilot sessions of an educational program were asked to complete the questionnaire draft. The program addressed early RRMS PwMS and consisted of a 4h interactive educational session and a 57-page EBPI booklet on diagnosis, prognosis and MS treatment [16]. We assessed the clarity and acceptability of the items, the response format and instructions, both quantitatively (analysis of responses provided) and qualitatively (interviews with participants immediately after questionnaire completion).

### Validation study

After minor revision (ordering of items, clarification of language) the RIKNO 1.0 questionnaire was used as an outcome measure in the PEPADIP RCT assessing the effectiveness of the above mentioned intervention in comparison to a stress management program in 192 early RR PwMS [16]. Construct validity was assessed by uni- and multivariate linear regression analyses considering the following characteristics: demographic (sex, age), education (primary, secondary school, university degree), clinical (disease courses; self-assessed disability–the UK Neurological Disability Scale [17], mood–the Hospital Anxiety and Depression Scale (HADS) [18, 19], locus of control, Kontrollüberzeugungen zu Krankheit und Gesundheit (KKG) [20] and role preferences in medical decisions, the Control Preference Scale [21]. The underlying hypothesis was that younger, better educated, less disabled PwMS with less anxiety and depression would show higher knowledge levels.

### Statistical analysis

To create a quality coefficient integrating measurement properties and patient relevance, the original 197 items were classified on both dimensions, which were standardized using z-scores. Items were ranked based on their quality coefficient. Those with moderate difficulty and high relevance were considered of high quality (Table 1). This quality coefficient was calculated separately for three patient groups (early RRMS, RRMS, SPMS) based on values generated in the corresponding subsamples while the PPMS cohort was too small to justify further analysis. Accordingly, a predefined algorithm (choosing the 4 highest ranked items of each domain) to compose a questionnaire draft, led to three different sets of items.

**Table 1:** Characteristics of the three patient groups who participated in RIKNO 1.0 validation.

	Item bank survey (n = 77)	Pilot study (n = 34)	PEPADIP RCT (n = 192)
	No (%)		
Women	55 (71%)	26 (76%)	142 (74%)
Age (years)*	45.0 (24–73)	39.6 (27–48)	36.6 (18–70)
Time since diagnosis (years)*	9.9 (0–34)	5.3 (1–28)	1.3 (0–2)
Disease course:**			
Early RRMS	22 (28%)	4 (12)	160 (83%)
RRMS	24 (31%)	20 (61)	0
SPMS	25 (34%)	9 (27)	0
PPMS	3 (4%)	0	0
Unclear	3 (4%)	1	32 (16%)
Education:			
University degree	40 (54%)	—	44 (23%)
Secondary school	28 (38%)	31***	99 (52%)
Primary school	6 (8%)	2	48 (25%)
Missing data	3	1	1
Immunotherapy (previous or ongoing)	19 (25%)	24	86 (45%)

*Mean (range).

** RRMS = relapsing remitting multiple sclerosis; SPMS = secondary progressive multiple sclerosis; PPMS = primary progressive multiple sclerosis.

***secondary school or higher.

The normality of the distribution of RIKNO 1.0 score was tested using the Kolmogorov-Smirnov test. Moreover, item difficulty was ascertained defining the range between 0.2 and 0.8 as appropriate. Adequate standard deviation was defined as values above 0.2.

Responsiveness was assessed by comparing mean total RIKNO 1.0 scores before and after the educative intervention, using paired t-tests in the pilot study and differences between intervention and control group using unpaired t-tests in the RCT. We explored the effect of general and clinical characteristics, role preference, and control belief characteristics on risk knowledge (RIKNO 1.0 total score) in the PEPADIP RCT sub-sample (baseline data) using linear regression. All the characteristics significantly associated with risk knowledge in univariate analysis were entered in the multivariate regression model.

Analyses were performed with SPSS version 15.1. All statistical tests were two-tailed and considered significant at an alpha level <0.05.

## Results

### Item bank

The expert panel identified a pool of 197 items, 19 of which were adopted from the previously developed tool [12]. The items covered the following domains: MS diagnosis (24 items, referring to 2 sub-domains: diagnosis, and diagnostic tests), course and prognosis (41 items, 4 sub-domains: disease course, course types, walking ability, life expectancy), MS in general (52 items, 7 sub-domains: MS definition, risk factors, symptoms, stress, relapses, quality of life, Expanded Disability Status Scale [22], therapies (51 items, 5 sub-domains: MS treatment in general, immunotherapy, evidence of immunotherapy effectiveness, pregnancy, rehabilitation), and evidence-based medicine (EBM) (29 items, 4 sub-domains: absolute and relative risk, RCTs, blinding, meta-analysis).

### Item selection and questionnaire construction

Seventy-seven of 120 (65%) questionnaires were returned and analyzed. Most participants had RRMS, with a mean disease duration of about 10 years (Table 1).

Data on item difficulty are reported in Table 2. Fourteen items were regarded as too difficult (i.e. scores < 0.20): six belonged to the domain ‘MS course/prognosis’, seven to ‘MS treatment’, one to ‘MS in general’. Forty-five items were too easy (score > 0.80): 15 belonged to the ‘MS treatment’ domain, nine to ‘MS in general’, eight to ‘MS course/prognosis’, seven to ‘EBM’ and six to ‘MS diagnosis’. The remaining 138 items were of intermediate difficulty.

**Table 2:** Distribution of difficulty and relevance in n = 77 PwMS participating in the 197-item bank survey.

Domain	No of items	Difficulty	Relevance
Diagnosis	24	0.53 (0.23)	1.94 (0.26)
MS course/ prognosis	52	0.56 (0.20)	1.95 (0.22)
MS in general	30	0.61 (0.28)	2.00 (0.27)
Evidence-based medicine	24	0.57 (0.25)	1.60 (0.45)
MS treatment	67	0.58 (0.24)	2.00 (0.24)

Data as mean (SD).

Item relevance was in general rated high (Table 2), with a mean value of 1.92 (SD 0.31, empirical range 0.81–2.5). Item difficulty and relevance were mainly unrelated, with an exception in the domain ‘MS in general’ (Spearman’s r 0.72, p<0.001). EBM items were rated as the least relevant. Judgements on relevance did not differ between patient groups (early MS, RRMS, SPMS and PPMS). However, according to the quality ranking, the ranking of items turned out different between patient groups.

The questionnaire draft for RRMS was based on data from the 22 early RR PwMS of the initial survey (Table 1). The subsets for the other patient groups were used to set up additional questionnaires. The resulting 20 single items were then organized by replacing the original yes/no response questions with multiple choice replies. In this transformation some of the top four ranked items were integrated in one multiple choice item. In addition, “distractor” item responses were included to increase difficulty, leading to a set of 14 multiple choice questions (4 on ‘MS in general’ and ‘Treatment’, 3 on ‘Diagnosis’, 2 on ‘EBM’, and one on ‘Prognosis’). Five additional items were added based on discussion by the expert panel leading to a total of 19 items for the final tool (see S1 File for the final RIKNO 1.0 questionnaire).

### Pilot study

Within the four pilot sessions, 34 questionnaires were completed (Table 1). Mean knowledge score was 9.8 (SD 3.2, range 0–16) with a minimum score of 0 and a maximum of 16. Item difficulty ranged from 0.18 to 0.85 (mean 0.50, SD 0.06). Overall, after the educational intervention PwMS knowledge increased by 2.44 points. Items were considered acceptable by participants. Although overall mean scores indicated that in general only 10 out of 19 questions were answered correctly, no further changes were performed.

### Validation study

In the PEPADIP RCT (n = 192) mean time since first symptoms was 4 years and 74% had an early RRMS course (Table 2). Mean RIKNO 1.0 score was 10.2 (SD 2.8; range 0–16). Normal distribution criteria were not met (Kolmogorov-Smirnov p = 0.004). Item difficulty was 0.58 on average, and ranged from 0.14 to 0.79. Item 5 on prognosis of untreated clinically isolated syndrome (answered correctly by 9%) and item 17 on prognostic indicators (answered correctly by 14%) were the most difficult (Table 3).

**Table 3:** Item difficulty of RIKNO 1.0 questionnaire in n = 192 PwMS (PEPADIP RCT).

Item no.	Domain	Mean (SD) difficulty
	**MS in general**	
1	MS definition (nerves and cells)	0.33 (0.47)
2	MS definition (nerve sheaths)	0.70 (0.46)
3	MS definition (nerve conduction)	0.40 (0.49)
4	MS definition (inflammation)	0.89 (0.31)
	**MS Diagnosis**	
9	Diagnosis (symptoms & images)	0.62 (0.49)
10	Meaning of MRI findings (course, relapse prediction)	0.67 (0.47)
13	Meaning of MRI findings (disability, degeneration)	0.26 (0.44)
14	Diagnosis (CSF, differential)	0.38 (0.48)
	**Prognosis**	
5	Disease course (MRI, relapses)	0.09 (0.29)
6	Benign MS	0.60 (0.49)
11	clinical prognostic markers (symptoms, course, gender)	0.65 (0.48)
12	Clinical prognostic markers (5 year evolution)	0.74 (0.48)
17	CIS relapse prognosis	0.14 (0.35)
	**Treatment**	
7	Exercise and MS	0.89 (0.31)
8	Steroids and relapses	0.55 (0.50)
18	IFN effect in CIS (ARR)	0.28 (0.45)
19	IFN effect in CIS (relapses)	0.93 (0.53)
	**Evidence-based medicine**	
15	RCTs	0.48 (0.50)
16	Evidence and RCTs	0.32 (0.47)

MS = multiple sclerosis; MRI = magnetic resonance imaging; CSF = cerebrospinal fluid; IFN = interferon; CIS = clinically isolated syndrome; ARR = absolute risk reduction; RCT = randomized controlled trial.

Items 19 (relapse frequency during early interferon treatment), 4 (MS as an inflammatory disease) and 7 (exercise as a putative disease modifying intervention) turned out to be the easiest questions, answered correctly by about 90%.

Two weeks after the educational mean RIKNO 1.0 score increased from 10.6 to 12.4 in the intervention group and only from 9.4 to 10.1 in the control group (between group comparison, p = 0.0003). The absolute difference of mean increases between intervention and control group was 1.1 correct answers.

### Construct validity (see Table 4)

In univariate linear regression analysis, characteristics significantly associated with higher risk knowledge were younger age (p = 0.002), higher education (p = 0.005), RR course (p = 0.002) and passive role preference at the CPS scale (p = 0.01) (Table 4). Variables retaining an independent effect in the multivariate model were younger age (p = 0.01) and passive role preference (p = 0.04).

**Table 4:** Characteristics associated to MS knowledge (RIKNO 1.0 total score) in 192 PwMS, (PEPADIP RCT sub-sample, baseline data) in linear regression analysis.

	Univariate			Multivariate		
Characteristic	Beta	P	R^2*^	Beta	P	R^2^ *
HADS-Anxiety score	-0.03	0.67	0.00 (-0.00)			
HADS-Depression score	-0.05	0.47	0.00 (-0.00)			
UNDS	-0.05	0.46	0.00 (-0.00)			
Age (years)	-0.22	**0.002**	0.05 (0.04)	-0.18	**0.01**	
Disease duration (years)	0.14	0.09	0.02 (0.01)			
Walking distance (meters)	0.11	0.22	0.01 (0.00)			
Women	0.07	0.31	0.01 (0.00)			
Early MS	-0.11	0.15	0.01 (0.01)			
Relapsing MS	0.22	**0.002**	0.05 (0.05)	0.17	0.07	
Chronic progressive MS	-0.18	**0.015**	0.03 (0.03)	-0.041	0.66	
Higher education	0.21	**0.005**	0.04 (0.04)	0.098	0.19	
Autonomous role preference(CPS scale)	-0.19	**0.01**	0.04 (0.03)	-0.15	**0.04**	
Locus of control (KKÜ sub-scales)	-0.04–0.030.05	0.590.670.51	0.00 (-0.00)0.00 (-0.01)0.00 (-0.00)			
Total					**<0.001**	0.14 (0.12)

HADS = Hospital anxiety and depression scale (19), UNDS = UK Disability Scale (17), CPS = Control Preference Scale (21), KKÜ = Kontrollüberzeugungen zu Gesundheit und Krankheit (20).

* corrected R^2^

## Discussion

A risk knowledge questionnaire for RRMS was developed based on a large item bank, taking into account item difficulty and patients’ perception of relevance. Selection of items with a moderate item difficulty and high perceived relevance led to a core set of 19 items which were framed using multiple choice response formats and supplemented with ‘distractor’ response items. The final tool showed an acceptable score distribution. Approximately half of the 19 items were answered correctly in the pilot study as well as in the PEPADIP RCT at baseline [16]. After exposure to an educational intervention (four-hour education programme and 57-page booklet) a small but significant increase in knowledge score could be detected. Still, on average seven items were not answered correctly after the intervention.

Does this indicate that the questionnaire had been insufficiently developed and/or is too difficult? While developing a risk knowledge questionnaire several aspects need to be addressed. The most important questions are: ‘Who decides about the relevance of risk knowledge?’ and ‘At what time point during the disease course should relevant risk knowledge be present among patients?’ For a patient with a MS diagnosis years ago, stable on an immunomodulatory drug relevance is substantially low. The time point when a decision about immunotherapy should be made seems the most relevant situation to assess risk knowledge. But relevance depends on the individual situation of the patient and especially from disease activity. Also at the stage of still unexplained symptoms suggestive of MS, there is a need for risk information [23]. Risk knowledge is here relevant to make a decision about diagnostic testing. Any risk questionnaire can only ask for aspects which might be more or less relevant for most patients.

But why not let patients judge on relevance? Although in general to be agreed on, there are a couple of problems with this approach. Patients might not be aware of the background of many of the uncertainties in diagnosing MS, to give a prognostic estimate and about the long term efficacy of treatments. They therefore might simply reject the relevance of items based on lack of knowledge. The most provocative example might be questions on absolute risk reductions which have been claimed to be at the core of risk knowledge [2, 24, 25]. These items were not considered highly relevant by patients and the questions on relapse frequency in 100 patients without and with treatment were only answered correctly by a minority of patients (about 30%).

In earlier work, patients have demonstrated their ability to understand and deal with absolute risk numbers after exposure to a tutorial [13]. This touches the difficulty in the general population to handle probabilistic information [26]. Communication of probabilistic information is an essential content in EBPI [13]. According to this concept patients should acquire a basic understanding of the natural history (i.e. placebo arms in treatment studies), event rates and especially absolute risk reductions (ARR) of treatment to personally appraise the value of their choices [13]. We believe this understanding is crucial as it is a major step towards appraisal of equipoise: for a given patient there is not one ideal treatment, and all the available options (including the option of not starting/deferring a treatment) need to be discussed [27]. Clinical equipoise is a fundamental ethical principle, and it is at the core of shared decision making [27]. By understanding the concept of equipoise and having correctly framed probabilistic information such as ARR, it is expected that patients are enabled to decide based on their own values and preferences, and not just to comply with physicians’ preferred options. Thereby, understanding this issue may lead to a change in health behaviour, not simply to increased knowledge.

Should therefore experts decide what is relevant? We believe the development of a core MS risk knowledge should be based on expert and patient input.

Construct validity analysis by regression did not show any impact of education, and a better knowledge in younger PwMS. Moreover, PwMS who preferred a passive role had higher knowledge, which is at odds of what can be expected. However, we do not think that this finding strongly questions validity as most items are highly MS specific and higher education might not have helped in answering. In addition difficulty of the questionnaire was high, which might have reduced the impact of the moderator variables. One might argue that showing responsiveness of the questionnaire in an education intervention while the intervention itself claimed efficacy through changes in the risk knowledge questionnaire is a tautologic. It should be noted that the questionnaire was developed independently from the intervention. So for example questions on exercise effects, relapses, efficacy of drugs in relapsing MS are not specifically tackled by the education intervention but are part of the questionnaire. This might be another explanation that the effects of the programme on RIKNO 1.0 are relatively small, nevertheless statistically significant in a cohort of 192 patients. Therefore we believe that RIKNO 1.0 assesses relevant risk knowledge and is sensitive to change. To avoid just confronting patients with their ignorance, correct answers should be communicated to them, or education suggested. Focus group meetings on the questionnaire conducted in PwMS from five European countries show highly engaged patient discussions around the topics. In some instances, PwMS moved from a feeling of guiltiness to a desire to explore treatment options and health care engagement.

Improving risk knowledge may alter PwMS health behaviour with impact on treatment uptake and adherence as we have shown in the area of relapse management and decisions on early immunotherapy [16, 28]. However, up to now it can only be assumed that these behavioural changes will impact on health outcomes.

To our knowledge, the only other existing MS knowledge questionnaire is the MSKQ [9] which has recently been translated in UK English, German, Dutch, and linguistically validated in French for France. The MSKQ has been also developed based on expert-derived items, and proved to be well responsive [10, 11], nevertheless it is much easier, so that about 64% of questions can be answered correctly by patients even without any educational intervention [10]. Furthermore, the tool does not specifically focus on risk knowledge or critical appraisal of treatment trial data. Therefore, the MSKQ provides rather a general estimate of MS knowledge, but does not seem to be the most suitable outcome tool for an EBPI intervention.

The current version of RIKNO 1.0 has been developed in the setting of one MS center in Germany, with subsequent assessment at German universities and specialized MS centers. This might have led to some selection bias. A non-representative percentage of only 25% of patients participating in the initial survey reported actual immunotherapy, indicating even more clearly an overrepresentation of treatment critical respondents (around 50% PwMS are on immunotherapy at the Hamburg center) [29]. However, in the PEPADIP RCT we found no differences between patients from the Hamburg center and other centers in Germany indicating that the long-standing attitude in the Hamburg center focussing EBPI has not led to substantial differences in knowledge levels in early MS patients [16].

Further work, already started in the AutoMS project (www.automsproject.org), discusses the questionnaire in expert and patient focus group meetings from different European countries in order to develop an harmonized international version of the tool and to compare performance with the other established measure, the MS knowledge questionnaire [9]. This internationally agreed upon risk knowledge tool for early MS might then be applicable not only as an outcome tool for educative intervention, but also as an estimate of the knowledge stage of patients in a given center. Nevertheless such a tool will need continued updating at best embedded in an international consensus platform.

## Supporting Information

S1 File (DOCX)
