# Supplementary material for: Risk Knowledge in Relapsing Multiple Sclerosis (RIKNO 1.0) - Development of an Outcome Instrument for Educational Interventions
Source: PLoS One. 2015 Oct 2;10(10):e0138364. doi: 10.1371/journal.pone.0138364 (PMC4591974; doi:10.1371/journal.pone.0138364)
Supplement: S1 File — (DOCX) [file pone.0138364.s001.docx]

# S1 File

**Risk knowledge questionnaire for RRMS**

**G=general question, D=diagnosis, P=prognosis, T=treatment, E=EBM**

**Please tick**

|  |  | |  | |
| --- | --- | --- | --- | --- |
|  |  |  | **Correct** | **Wrong** |
|  | MS is a disease of the central nervous system where nerve fibers and nerve cells are destroyed. | | X | □ |
|  | |  | | |
|  | MS is a disease of the central nervous system where nerve sheaths are destroyed. | | X | □ |
|  | |  | | |
|  | MS is a disease of the nervous system disease where peripheral nerve conduction to muscles is disturbed. | | □ | x |
|  | |  | | |
|  | MS is an inflammatory disease of the central nervous system. | | X | □ |

= all G

**Please tick (only one):**

| 1. **P** | **Which of the following statements is wrong?** |  | |
| --- | --- | --- | --- |
|  | MS disease course is possibly related to the number of inflammatory lesions on a first MRI. | □ | |
|  | The extent of residual deficit during MS disease course is higher when many relapses occur. | x | |
|  | MS disease course is possibly related to severity of and recovery from a first relapse. | □ | |
|  | MS disease course depends on age at disease onset. | □ | |
| 1. **P** | **Which of the following statements is correct?** | |  |
|  | There is no benign MS. | | □ |
|  | Possibly up to 30% of disease courses are benign which means that patients will have no difficulties in activities of daily living after 20 years. | | x |
|  | The rate of benign MS courses can be estimated easily from the available studies. | | □ |
|  | The percentage of benign courses has not changed during the last 30 years. | | □ |

| 1. **T** | **Which of the following statements is wrong?** |  |
| --- | --- | --- |
|  | Possibly, sports have beneficial effects on cognitive performance in MS. | □ |
|  | Sports impact on the immune system of MS patients. | □ |
|  | Sports can improve mobility and quality of life in MS. | □ |
|  | Sports can negatively influence the course of MS. | x |

| 1. **T** | **Which of the following statements is correct?** |  |
| --- | --- | --- |
|  | Steroid treatment in acute relapses can shorten relapse duration. | x |
|  | Steroid treatment in acute relapses can prevent further relapses. | □ |
|  | Steroid treatment in acute relapses can slow down the progression of MS. | □ |
|  | Steroid treatment in acute relapses can lead to less sustained disability compared to no treatment. | □ |

| 1. **D** | **Which of the following statements is wrong?** |  |
| --- | --- | --- |
|  | First-time typical symptoms are sufficient to make a MS diagnosis. | x |
|  | MS diagnosis can be made if typical symptoms and additional typical MRI findings are present. | □ |
|  | Diagnosis of MS can be made based on the disease course. | □ |
|  | Since 2011 a MS diagnosis is possible after only one relapse. | □ |

| 1. **D** | **Which of the following statements is wrong?** |  |
| --- | --- | --- |
|  | The number of lesions on MRI at any time point does not predict the further disease course. | □ |
|  | A contrast enhancing lesion on MRI is predictive for an upcoming relapse. | X |
|  | MRI with contrast agents does not make sense shortly after a steroid course. | □ |
|  | MRI mirrors the inflammatory activity of MS. | □ |

| 1. **P** | **Which of the following statements is correct?** |  |
| --- | --- | --- |
|  | The course of MS depends on the presentation of first symptoms. | □ |
|  | Development of disability depends on the disease course at the beginning (relapsing or progressive MS). | x |
|  | In MS sooner or later always residual deficits will develop. | □ |
|  | The MS disease course depends on gender. | □ |

| 1. **P** | **Which of the following statements is correct?** |  |
| --- | --- | --- |
|  | The course of the disease can be predicted from the disabilities developed until the 5th year. | □ |
|  | Many relapses in the 5th year of MS point towards a severe disease course. | □ |
|  | From the number of inflammatory lesions on MRI the further disease course can be reliably predicted. | □ |
|  | A severe first relapse is no definite predictor for an unfavourable disease course. | x |

| 1. **D** | **Which of the following statements is wrong?** |  |
| --- | --- | --- |
|  | From MRI patient’s disability can be determined. | □ |
|  | Brain atrophy can only be demonstrated on MRI at late disease stages. | □ |
|  | Black holes on MRI show always destroyed nervous tissue. | x |
|  | Inflammatory lesions (white spots) on MRI show destruction of nerve sheaths and nerve cells. | □ |

| 1. **D** | **Which of the following statements is correct?** |  |
| --- | --- | --- |
|  | Without analysis of CSF no MS diagnosis can be made. | □ |
|  | There is a typical MRI pattern which clearly suggests MS. | □ |
|  | Tick-born diseases (borreliosis) and certain rheumatic diseases can clearly be separated from MS. | □ |
|  | Sometimes a definite MS diagnosis cannot be made even after many years. | x |

| 1. **E** | **Which of the following statements is wrong?** |  |
| --- | --- | --- |
|  | A double-blind, randomised, placebo-controlled trial proves for all MS patients if a drug is effective or not. | x |
|  | A double-blind, randomised, placebo-controlled trial is a study in which a drug is tested against a dummy drug (placebo). Physicans and patients do not know who receives the drug. Patients are randomly allocated to treatment groups. | □ |
|  | A double-blind, randomised, placebo-controlled trials often only gives evidence of efficacy for a highly selected patient group. | □ |
|  | Longitudinal observations of patients after termination of a double-blind, randomised-controlled trial cannot provide evidence for treatment efficacy. | □ |

|  | **E Which of the following statements is wrong?** |  |
| --- | --- | --- |
|  | The best evidence on the efficacy of MS drugs stems from expert statements as e.g. from the German competence network. | x |
|  | The best evidence for efficacy of drugs stems from double-blind, randomised, placebo-controlled trials. | □ |
|  | Even when a study has shown a significant result, it might be without value for treatment. | □ |
|  | Studies with few participants, which do not show an effect between a treatment and a placebo group might overlook an existing treatment effect. | □ |

|  | **Which number is correct?** | |
| --- | --- | --- |
|  | Imagine 100 patients with a first clinical episode of MS.  How many patients will not have a second relapse within 2 years without treatment? | □ 10 of 100  □ 20 of 100  □ 40 of 100  x 60 von 100 |
|  | Imagine 100 patients with a first clinical episode of MS.  How many patients will not have a second relapse within 2 years due to interferon treatment compared to patients who did not receive any treatment? | x 20 of 100  □ 40 of 100  □ 60 of 100  □ 80 of 100 |
|  | Imagine 100 patients with a first clinical episode of MS. All start an interferon treatment. How many relapses will statistically occur per patient within the next 2 years | □ 0 relapses  x 1-2 relapses  □ 2-4 relapses  □ 4 relapses |

= all 3 T
